# Metastatic Pattern Discriminates Survival Benefit of Type of Surgery in Patients With *De Novo* Stage IV Breast Cancer Based on SEER Database

**DOI:** 10.3389/fsurg.2021.696628

**Published:** 2021-11-03

**Authors:** Kunlong Li, Can Zhou, Yan Yu, Ligang Niu, Wei Zhang, Bin Wang, Jianjun He, Guanqun Ge

**Affiliations:** ^1^Department of Breast Surgery, First Affiliated Hospital, Xi'an Jiaotong University, Xi'an, China; ^2^School of Medicine, Xi'an Jiaotong University, Xi'an, China

**Keywords:** SEER, IPTW, *de novo* stage IV BC, surgery, metastatic patterns

## Abstract

**Background:** The role of surgery and surgery type in *de novo* stage IV breast cancer (BC) is unclear.

**Methods:** We carried out a retrospective cohort study that included the data of 4,108 individuals with *de novo* stage IV BC abstracted from SEER (Surveillance, Epidemiology, and End Results) data resource from 2010 to 2015. The patients were stratified into the non-surgery group, breast-conserving (BCS) surgery group, and mastectomy group. Inverse probability propensity score weighting (IPTW) was then used to balance clinicopathologic factors. Overall survival (OS), as well as the breast cancer-specific survival (BCSS), was assessed in the three groups using Kaplan–Meier analysis and COX model. Subgroups were stratified by metastatic sites for analysis.

**Results:** Of the 4,108 patients, 48.5% received surgery and were stratified into the BCS group (574 cases) and mastectomy group (1,419 cases). After IPTW balance demographic and clinicopathologic factors, BCS and mastectomy groups had better OS (BCS group: HR, 0.61; 95% CI: 0.49–0.75; mastectomy group: HR, 0.7; 95% CI: 0.63–0.79) and BCSS (BCS group: HR, 0.6; 95% CI, 0.47–0.75; mastectomy group: HR, 0.71; 95% CI, 0.63–0.81) than the non-therapy group. Subgroup analyses revealed that BCS, rather than mastectomy, was linked to better OS (HR, 0.66; 95% CI: 0.48–0.91) and BCSS (HR, 0.63; 95% CI: 0.45–0.89) for patients with bone-only metastasis. For patients with viscera metastasis or bone+viscera metastases, BCS achieved similar OS (viscera metastasis: HR, 1.05; 95% CI: 0.74–1.48; bone+viscera metastases: HR, 1.01; 95% CI: 0.64–1.61) and BCSS (viscera metastasis: HR, 0.94; 95% CI: 0.64–1.38; bone+viscera metastases: HR, 1.06; 95% CI: 0.66–1.73) in contrast with mastectomy.

**Conclusions:** Local surgery for patients with distant metastasis (DS) exhibited a remarkable survival advantage in contrast with non-operative management. BCS may have more survival benefits for patients with *de novo* stage IV BC with bone-only metastasis than other metastatic sites. Decisions on *de novo* stage IV BC primary surgery should be tailored to the metastatic pattern.

## Introduction

There is an ongoing epidemic of breast cancer (BC) among women all over the world, and to date, it is a universally acknowledged fact that this disease is the most frequent form of cancer Lands far and near (1, 2). About 3%−8% of BC cases are detected in stage IV (3), and BC with distant metastasis (DM) is generally incurable, with a median overall survival (OS) of 2–3 years (4, 5). Given its poor prognosis, treating primary tumors in *de novo* stage IV BC remains a vital position. Treatment aims to relieve the symptoms, enhance the quality of life (QOL), as well as prolong survival (6). Advancements in systemic treatment have remarkably improved metastatic disease control along with survival (7, 8). Nonetheless, the role of surgery and surgery type in *de novo* stage IV BC treatment is unclear, and the consensus is lacking.

Numerous retrospective studies have illustrated that local surgery improves the prognoses of patients with BC with DMs (9, 10). However, three prospective randomized trials have generated controversial findings. MF07-01 trial updated their data at a median follow-up of 40 months, and a remarkably different improvement in OS was observed in favor of performing surgery (11). However, the Indian Tata Memorial, as well as ABCSG-28 POSYTIVE trials, found no association between prognosis and surgery (12, 13). Moreover, some studies suggest that surgery may even accelerate metastatic growth, adversely affecting survival (14, 15). These inconsistent outcomes are attributed to differences in metastatic patterns, which affect prognosis (11, 16–18). Thus, individualized clinical strategies are needed for *de novo* stage IV BC.

Here, we explored the survival benefits of primary surgery and surgery scheme in *de novo* stage IV BC categorized by metastatic profiles. We followed a large cohort of *de novo* stage IV BC from the population-based SEER data resource (Surveillance, Epidemiology, and End Results) from 2010 to 2015.

## Materials and Methods

### Data Resource

The recent version of SEER 18 registries Custom Data (with additional treatment fields) was employed as a data resource for this retrospective longitudinal study. This database is comprised of 18 population-based cancer registries, representing about 26% of the USA population (19). SEER^*^-Stat V.8.3.8 (https://seer.cancer.gov/seerstat/) (Information Management Service, Inc.) was employed in generating case listing. The approved guidelines were followed in all the procedures. This study was granted approval by the ethics committee of the First Affiliated Hospital of Xi'an Jiaotong University. The consent of the participants is not required to access and use SEER data.

### Patient Cohort

Cases of 14,968 individuals who had been diagnosed with stage IV BC from January 1, 2010, to December 31, 2015, were identified in the SEER data resource. According to the SEER program, diagnosis of metastases in the first 4 months of diagnosis is defined as initial stage IV BC. The demographic along with the clinicopathologic variables contained sex (female), tumor T stage, age, tumor N stage, race, histology, tumor grade, radiotherapy, type of surgery, breast subtype, chemotherapy, survival months, the status of DS, vital status, cause of death, breast-adjusted American joint committee on cancer (AJCC) sixth tumor node metastasis (TNM) stage, and marital status.

After the first selection, participants were excluded based on the following criteria: (1) patients with multiple primary tumors, (2) follow-up autopsy type or death certificate, (3) not receiving any non-surgical treatment (chemotherapy or radiotherapy), (4) unknown metastatic sites, (5) unknown breast subtype, (6) aged <18 years old, (7) missing surgical records, (8) patients without metastasis or with brain metastasis, and (9) survival time of <6 months.

**Figure 1:**
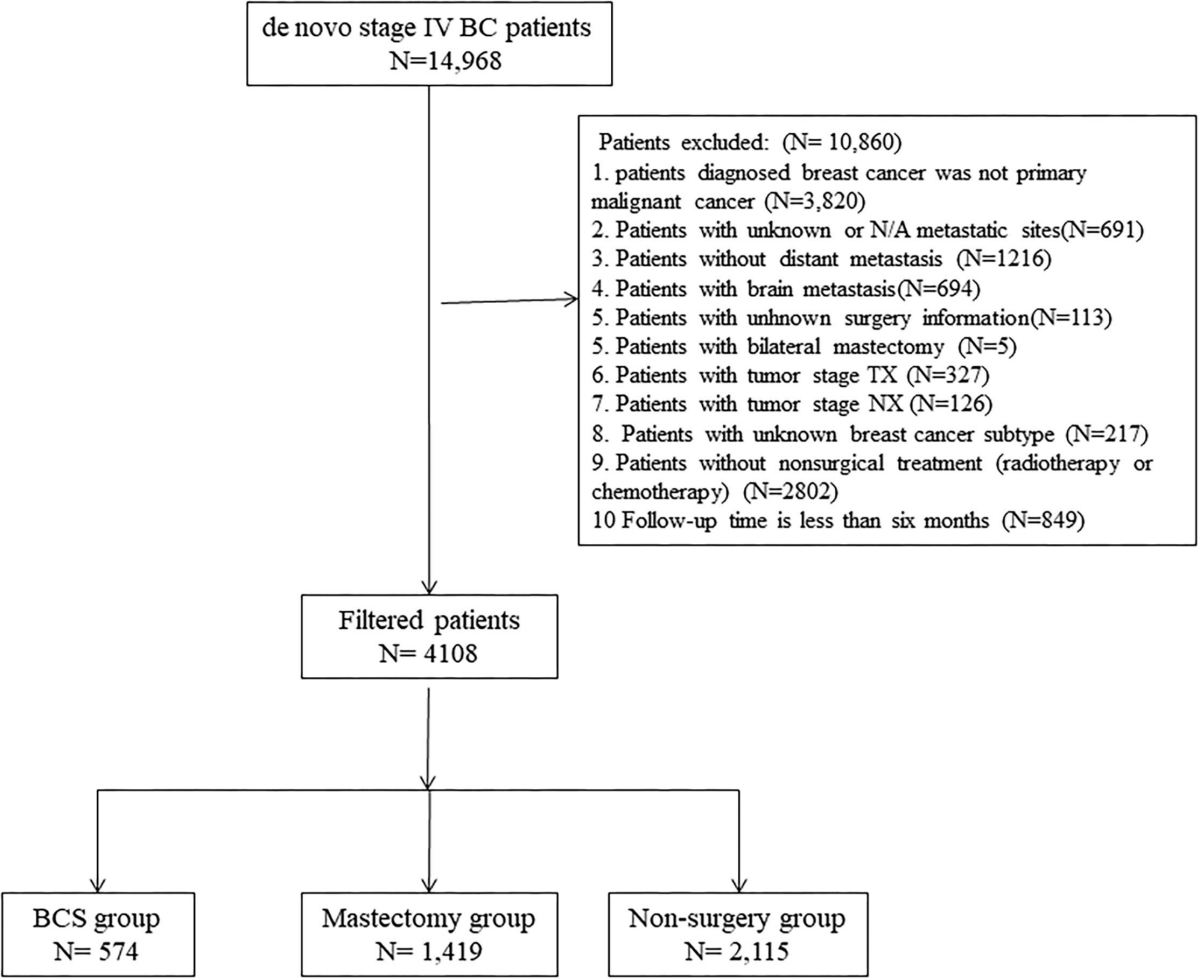
Eligibility, inclusion, and exclusion criteria of the study population.

About 4,108 patients with stage IV BC were enrolled. To estimate the impact of surgery on prognosis, the enrolled dataset was stratified into three groups based on operation selection: non-surgery group, breast-conserving surgery (BCS) group, and mastectomy group. Based on SEER Program Coding and Staging Manual, 2016, local tumor destruction, partial mastectomy, and subcutaneous mastectomy were regarded as BCS. Extended radical mastectomy, simple mastectomy, modified radical mastectomy, as well as radical mastectomy, were regarded as mastectomy. And “No radiation and/or cancer-directed surgery” was regarded as no radiotherapy. “No/Unknown” chemotherapy records were regarded as no chemotherapy. To evaluate surgical options for different metastatic sites, the patterns were categorized into bone-only, viscera, and bone+viscera. The screening process is outlined in Figure 1.

### Endpoints

The patients whose data were used in this study had been followed up until November 2015. OS was the primary index, which was defined as the time beginning the diagnosis date to the date of death due to any cause, while the secondary outcome measurements were breast cancer-specific survival (BCSS), defined as the time beginning the diagnosis date to the date of death from BC.

## Statistical Analysis

All statistical analyses were implemented in R V. 3.6.3 (https://www.r-project.org). Clinicopathologic and demographic factors were compared between non-surgery, BCS, and mastectomy groups with the chi-square test or Fisher's exact test, as appropriate. Inverse probability propensity score weighting (IPTW) (20–22) was employed to balance clinicopathologic and demographic characteristics among the above-mentioned groups. Propensity scores were calculated based on race, tumor grade, histology, tumor T stage, non-surgical treatment, tumor N stage, metastatic organs, breast subtype, age, and marital status using a generalized boosted model (GBM) for receipt of different surgeries (22, 23). Propensity score weighted log-rank tests along with Cox proportional hazard model were used to compare OS and BCSS among the three groups. OS and BCSS HR with 95% CI were determined from multivariable models corrected for baseline characteristics of the patients. Metastatic pattern subgroups were analyzed similarly.

## Results

### Baseline Characteristics

A total of 4,108 individuals with *de novo* stage IV BC were eligible for analyses. Of these, 51.5% were non-surgical. Of the 48.5% who received surgery, 574 and 1,419 belonged to the BCS and mastectomy groups, respectively. Of these patients, 54.5% had poorly differentiated or undifferentiated BC (grade III or IV), 82.9% had infiltrating duct carcinoma, 35.9% had T2 stage BC, 48.8% had N1 stage BC, 68% had received chemotherapy only, 45.4% had bone-only metastasis, 51.3% had Luminal A BC, 73.1% were white, and 48.6% were married. By comparing non-surgery (BCS) and mastectomy groups, remarkable differences (*p* = <0.05) were found in grade, stage, histology, T-stage, N-stage, non-surgical treatment, metastatic sites, molecular subtype, age, and marital status. Detailed information is shown in Table 1. Balance in patient features was attained after adjustments of the propensity score for predicting the average treatment impact (Table 2).

**Table 1:** The baseline characteristics of patients with different surgery procedures in the SEER database.

	**Total**	**BCS**	**Mastectomy**	**Non-surgery**	***P-value***

**ITEMS**	***N*** **(%)**	***N*** **(%)**	***N*** **(%)**	***N*** **(%)**	
	4,108 (100)	574 (14)	1,419 (34.5)	2,115 (51.5)	
**Age**	55.32 (13.05)	56.28 (12.79)	54.71 (13.28)	55.47 (12.94)	0.04
**Grade**					<0.001
I–II	1,869 (45.5)	255 (44.4)	574 (40.5)	1,040 (49.2)	
III–IV	2,239 (54.5)	319 (55.6)	845 (59.5)	1,075 (50.8)	
**Histology**					<0.001
Infiltrating duct carcinoma	3,404 (82.9)	490 (85.4)	1,126 (79.4)	1,788 (84.5)	
Other	704 (17.1)	84 (14.6)	293 (20.6)	327 (15.5)	
**T_stage**					<0.001
T1	443 (10.8)	112 (19.5)	102 (7.2)	229 (10.8)	
T2	1,474 (35.9)	321 (55.9)	481 (33.9)	672 (31.8)	
T3	811 (19.7)	70 (12.2)	319 (22.5)	422 (20.0)	
T4	1,380 (33.6)	71 (12.4)	517 (36.4)	792 (37.4)	
**N_stage**					<0.001
N0	718 (17.5)	152 (26.5)	150 (10.6)	416 (19.7)	
N1	2,005 (48.8)	237 (41.3)	571 (40.2)	1,197 (56.6)	
N2	628 (15.3)	100 (17.4)	328 (23.1)	200 (9.5)	
N3	757 (18.4)	85 (14.8)	370 (26.1)	302 (14.3)	
**Non-surgical treatment**					<0.001
Chemotherapy	2,795 (68.0)	225 (39.2)	659 (46.4)	1,911 (90.4)	
Radiotherapy	363 (8.8)	119 (20.7)	147 (10.4)	97 (4.6)	
Radiotherapy+Chemotherapy	950 (23.1)	230 (40.1)	613 (43.2)	107 (5.1)	
**Metastatic sites**					<0.001
Bone+viscera	1,110 (27.0)	91 (15.9)	251 (17.7)	768 (36.3)	
Bone_only	1,867 (45.4)	325 (56.6)	747 (52.6)	795 (37.6)	
Viscera	1,131 (27.5)	158 (27.5)	421 (29.7)	552 (26.1)	
**Molecular subtype**					0.001
HER2-enriched	489 (11.9)	57 (9.9)	174 (12.3)	258 (12.2)	
Luminal A	2,106 (51.3)	317 (55.2)	729 (51.4)	1,060 (50.1)	
Luminal B	948 (23.1)	117 (20.4)	293 (20.6)	538 (25.4)	
Triple-negative	565 (13.8)	83 (14.5)	223 (15.7)	259 (12.2)	
**Race**
White	3,004 (73.1)	430 (74.9)	1,041 (73.4)	1,533 (72.5)	0.49
Unwhite	1,104 (26.9)	144 (25.1)	378 (26.6)	583 (27.5)	
**Marital status**					0.009
Married	1,996 (48.6)	306 (53.3)	716 (50.5)	974 (46.1)	
Single	1,930 (47.0)	242 (42.2)	639 (45.0)	1,049 (49.6)	
Unknown	182 (4.4)	26 (4.5)	64 (4.5)	92 (4.3)	

*HER2, human epidermal growth receptor 2; BCS, breast-conserving surgery*.

**Table 2:** The baseline characteristics of patients with different surgery procedures in the SEER database after IPTW.

	**Total**	**BCS**	**Mastectomy**	**Non-surgery**	***P-value***

**ITEMS**	***N*** **(%)**	***N*** **(%)**	***N*** **(%)**	***N*** **(%)**	
	4,108	574	1,419	2,115	
**Age**	55.32 (13.05)	56.28 (12.79)	54.71 (13.28)	55.47 (12.94)	0.04
**Grade**					0.12
I–II	1,866 (45.4)	256 (44.6)	617 (43.5)	993 (46.9)	
III–IV	2,242 (54.6)	318 (55.4)	802 (56.5)	1,122 (53.1)	
**Histology**					<0.001
Infiltrating duct carcinoma	3,430 (83.5)	490 (85.4)	1,172 (82.6)	1,768 (83.6)	
Other	678 (16.5)	84 (14.6)	247 (17.4)	347 (16.4)	
**T_stage**					0.28
T1	427 (10.5)	64 (11.2)	141 (9.9)	222 (10.5)	
T2	1,439 (35)	221 (38.5)	499 (35.2)	719 (34)	
T3	803 (19.5)	98 (17)	294 (20.7)	411 (19.4)	
T4	1,439 (35)	191 (33.3)	485 (34.2)	763 (36.1)	
**N_stage**					0.51
N0	698 (17)	96 (16.7)	235 (16.6)	367 (17.4)	
N1	2,038 (49.6)	285 (49.6)	683 (48.1)	1,070 (50.6)	
N2	622 (15.1)	94 (16.3)	229 (16.2)	299 (14.1)	
N3	751 (18.3)	100 (17.4)	272 (19.2)	379 (17.9)	
**Non-surgical treatment**					0.003
Chemotherapy	2,845 (69.3)	378 (65.8)	945 (66.6)	1,522 (72)	
Radiotherapy	357 (8.7)	55 (9.5)	129 (9.1)	173 (8.2)	
Radiotherapy+Chemotherapy	907 (22)	142 (24.7)	345 (24.3)	420 (19.8)	
**Metastatic sites**					0.28
Bone+viscera	1,115 (27.1)	157 (27.4)	358 (25.2)	600 (28.4)	
Bone_only	1,835 (44.7)	249 (43.3)	646 (45.5)	940 (44.4)	
Viscera	1,158 (28.2)	168 (29.3)	415 (29.3)	575 (27.2)	
**Molecular subtype**					0.74
HER2-enriched	494 (12)	73 (12.7)	172 (12.1)	249 (11.8)	
Luminal A	2,052 (50)	274 (47.7)	713 (50.2)	1,065 (50.4)	
Luminal B	978 (23.8)	147 (25.7)	322 (22.7)	509 (24)	
Triple-negative	585 (14.2)	80 (13.9)	213 (15)	292 (13.8)	
**Race**					0.6
White	3,014 (73.4)	431 (75.1)	1,038 (73.1)	1,545 (73.1)	
Unwhite	1,094 (26.6)	143 (24.9)	381 (26.9)	570 (26.9)	
**Marital status**					0.71
Married	2,010 (48.9)	290 (50.4)	703 (49.5)	1,017 (48.1)	
Single	1,910 (46.7)	259 (45.1)	650 (45.9)	1,010 (47.7)	
Unknown	181 (4.4)	26 (4.5)	66 (4.6)	89 (4.2)	

*HER2, human epidermal growth receptor 2; IPTW, inverse probability propensity score weighting; BCS, breast-conserving surgery*.

### Kaplan–Meier Analysis of OS and BCSS After IPTW

**Figure 2:**
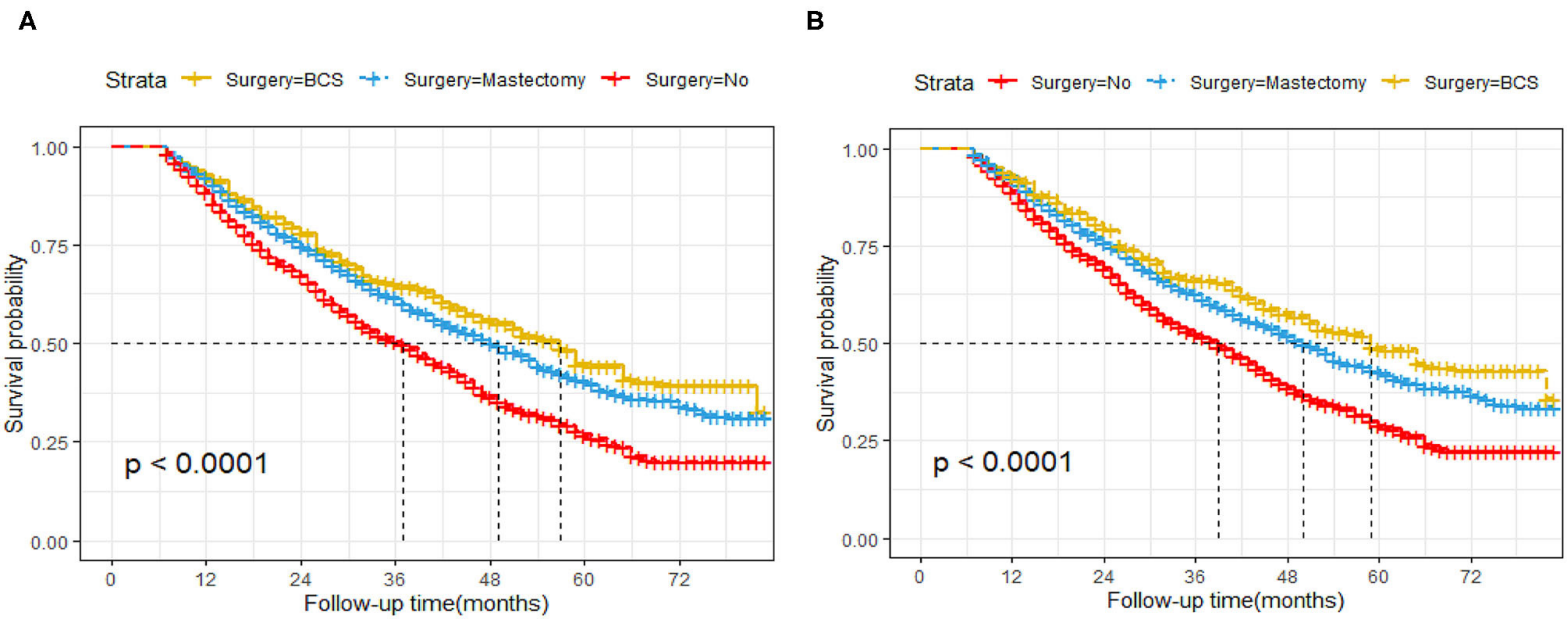
Kaplan–Meier survival analysis for patients with *de novo* stage IV Breast Cancer after IPTW. **(A)** Overall survival curves. **(B)** Breast cancer-specific survival curves.

About 51.3% (1,941/4,108) of the patients in this cohort study died after a median follow-up time of 27 months from diagnosis. Of these, 91.3% (1,771/1,941) were BC-specific deaths, while 8.7% (170/1,941) were due to other causes. After weighing inverse propensity score, the 3 year OS rate was 50.4, 65, and 61.5% in the non-surgery, BCS, and mastectomy group, respectively. The 5 year OS rate was 26.8, 44.6, and 40.5% in the non-surgery, BCS, and mastectomy group, respectively. The 3 year BCSS rate was 52.3, 66.3, and 62.7% in the non-surgery, BCS, and mastectomy group, respectively. The 5 year BCSS rate was 29, 48.8, and 42.9% in the non-surgery, BCS, and mastectomy group, respectively. Compared to non-surgery patients, BCS and mastectomy recipients had significantly higher OS (BCS group: 95% CI: 0.49–0.75, *p* = <0.001, HR, 0.61; mastectomy group: HR, 0.7, 95% CI: 0.63–0.79, *p* = <0.001) and BCSS (BCS group: HR, 0.6, 95% CI: 0.47–0.75, *p* = <0.001; mastectomy group: HR, 0.71, 95% CI: 0.63 0.81, *P* < 0.001) in patients with stage IV BC (Figures 2A,B).

### Univariate Along With Multivariate Cox Regression Model Analysis of MaBC Patients After IPTW

Univariate Cox analysis revealed that age, tumor grade, race, T-stage, type of surgery, non-surgical treatment, molecular subtype, metastatic pattern, and marital status were remarkably linked to OS and BCSS (Table 3). To identify independent predictors for OS and BCSS, multivariate Cox proportional hazard regression analysis was conducted. After adjusting clinical factors and considering propensity score in the Cox proportional hazard regression models, we found that relative to non-surgery patients, patients in BCS group and mastectomy group exhibited better OS (BCS group: HR, 0.59, 95% CI: 0.47–0.75, *p* = <0.001; mastectomy group: *p* = <0.001, HR, 0.68, 95% CI: 0.59–0.77) and BCSS (BCS group: HR, 0.58, 95% CI: 0.45–0.75, *p* = <0.001; mastectomy group: HR, 0.69, 95% CI: 0.6–0.79, *p* = <0.001). Additionally, age, grade, tumor grade, T-stage, non-surgical treatment, metastatic pattern, and molecular subtype were also independent predictive factors for OS and BCSS.

**Table 3:** Multivariate analysis of prognostic factors of BCSS and OS in metastatic breast cancer after IPTW.

	**OS**						**BCSS**					
**ITEMS**	**Univariate analysis**			**Multivariate analysis**			**Univariate analysis**			**Multivariate analysis**		
	**HR**	**95% CI**	***P-value***	**HR**	**95% CI**	***P-valu*e**	**HR**	**95% CI**	***P-value***	**HR**	**95% CI**	***P-valu*e**
Age	1.01	1.008–1.02	<0.001	1.01	1.002–1.02	0.005	1.02	1.01–1.03	<0.001	1.02	1.01–1.03	0.02
**Grade**
I–II	As reference			As reference			As reference			As reference		
III–IV	1.69	1.46–1.95	<0.001	1.58	1.34–1.86	<0.001	1.75	1.5–2.04	<0.001	1.63	1.38–1.94	<0.001
**Histology**
Infiltrating duct carcinoma	As reference						As reference					
Other	0.98	0.81–1.18	0.8				1.01	0.83–1.23	0.96			
**T_stage**
T1	As reference			As reference			As reference			As reference		
T2	1.26	1.01–1.58	0.04	1.21	0.96–1.52	0.11	1.32	1.04–1.67	0.02	1.26	0.99–1.6	0.06
T3	1.55	1.21–1.99	<0.001	1.31	1.02–1.69	0.04	1.66	1.29–2.16	<0.001	1.39	1.07–1.81	0.01
T4	1.82	1.43–2.33	<0.001	1.51	1.18–1.94	0.001	1.91	1.47–2.48	<0.001	1.57	1.21–2.04	0.001
**N_stage**
N0	As reference						As reference					
N1	1.07	0.88–1.32	0.5				1.1	0.89–1.35	0.38			
N2	1.27	0.99–1.61	0.05				1.3	1–1.68	0.05			
N3	1.17	0.93–1.46	0.18				1.2	0.94–1.53	0.14			
**Type of surgery**
Non-surgery	As reference			As reference			As reference			As reference		
BCS	0.61	0.49–0.75	<0.001	0.59	0.47–0.75	<0.001	0.6	0.47–0.75	<0.001	0.58	0.45–0.75	<0.001
Mastectomy	0.7	0.63–0.79	<0.001	0.68	0.59–0.77	<0.001	0.71	0.63–0.81	<0.001	0.69	0.6–0.79	<0.001
**Non-surgical treatment**
Chemotherapy	As reference			As reference			As reference			As reference		
Radiotherapy	0.86	0.71–1.04	0.11	0.99	0.79–1.25	0.97	0.87	0.71–1.07	0.18	1.03	0.81–1.31	0.83
Radiotherapy+Chemotherapy	0.8	0.67–0.94	0.007	0.84	0.7–1.01	0.07	0.79	0.67–0.94	0.01	0.84	0.69–1.02	0.08
**Metastatic pattern**
Bone+viscera	As reference			As reference			As reference			As reference		
Bone_only	0.55	0.45–0.66	<0.001	0.55	0.46–0.66	<0.001	0.53	0.44–0.63	<0.001	0.54	0.45–0.65	<0.001
Viscera	0.83	0.68–1.01	0.06	0.68	0.55–0.84	<0.001	0.81	0.66–1.01	0.05	0.67	0.54–0.84	<0.001
**Molecular subtype**
HR–/HER2– (Triple-negative)	As reference			As reference			As reference			As reference		
HR–/HER2+ (HER2-enriched)	0.26	0.2–0.36	<0.001	0.27	0.2–0.36	<0.001	0.26	0.19–0.36	<0.001	0.27	0.2–0.36	<0.001
HR+/HER2– (Luminal A)	0.32	0.27–0.39	<0.001	0.41	0.33–0.51	<0.001	0.32	0.27–0.39	<0.001	0.42	0.33–0.52	<0.001
HR+/HER2+ (Luminal B)	0.21	0.16–0.27	<0.001	0.21	0.17–0.28	<0.001	0.2	0.16–0.27	<0.001	0.21	0.16–0.27	<0.001
**Race**
Unwhite	As reference			As reference			As reference			As reference		
White	0.82	0.71–0.95	0.008	0.93	0.79–1.09	0.37	0.83	0.71–0.97	0.02	0.94	0.79–1.12	0.51
**Marital status**
Married	As reference			As reference			As reference			As reference		
Single	1.24	1.07–1.44	0.005	1.12	0.96–1.31	0.14	1.24	1.06–1.45	0.01	1.14	0.97–1.34	0.12
Unknown	1.16	0.87–1.54	0.3	1.1	0.83–1.45	0.51	1.13	0.83–1.52	0.45	1.07	0.8–1.43	0.66

*BCSS, breast cancer-specific survival; OS, overall survival; HR, hazard ratio; CI, confidence interval; HER2, human epidermal growth receptor 2; BCS, breast-conserving surgery; IPTW, inverse probability propensity score weighting*.

### Subgroup Analysis After IPTW

To explore the influence of metastatic pattern on the choice of surgical strategy for *de novo* stage IV BC, subgroup analyses were performed after IPTW (Tables 4, 5). This analysis showed that relative to mastectomy recipients with bone-only metastasis, BCS recipients had better OS (95% CI: 0.48–0.91; *p* = <0.001; HR, 0.66) and BCSS (*p* = 0.01; HR, 0.63; 95% CI, 0.45–0.89), while non-surgery patients had poorer OS (HR, 1.73; 95% CI, 1.4–2.14; *p* = 0.01) and BCSS (HR, 1.65; 95% CI, 1.33–2.06; *p* = <0.001). Moreover, BCS recipients had similar OS relative to mastectomy recipients with viscera metastasis or bone+viscera metastases (viscera metastasis: HR, 1.05, *p* = 0.81; 95% CI, 0.74–1.48, bone+viscera metastases: HR, 1.01, 95% CI, 0.64–1.61, *p* = 0.96) and BCSS (viscera metastasis: HR, 0.94, 95% CI, 0.64–1.38, *p* = 0.75; bone+viscera metastases: 95% CI, 0.66–1.73, *p* = 0.8, HR, 1.06,), while non-surgery patients had worse OS (viscera metastasis: HR, 1.35, 95% CI, 1.06–1.73, *p* = 0.02; bone+viscera metastases: HR, 1.33, *p* = 0.02, 95% CI, 1.04–1.7) and BCSS (viscera metastasis: HR, 1.32, *p* = 0.04, 95% CI, 1.02–1.7,; bone+viscera metastases: HR, 1.37, *p* = 0.02, 95% CI, 1.06–1.77).

**Table 4:** Multivariate analysis of prognostic factors of OS for specific sites of metastases after IPTW.

**Metastatic sites**	**Only_bone**			**Viscera**			**Bone+viscera**		
	**HR**	**95% CI**	***P-value***	**HR**	**95% CI**	***P-valu*e**	**HR**	**95% CI**	***P-value***
Age	1.01	1.005–1.02	0.04	1.01	0.99–1.02	0.06	1.01	1.002–1.02	0.02
**Grade**
I–II	As reference			As reference			As reference		
III–IV	1.37	1.11–1.69	0.003	1.66	1.23–2.23	<0.001	1.66	1.24–2.23	<0.001
**T_stage**
T1	As reference			As reference					
T2	1.52	1.05–2.22	0.03	1.06	0.73–1.55	0.74			
T3	1.87	1.25–2.79	0.002	1.06	0.71–1.59	0.78			
T4	1.85	1.25–2.74	0.002	1.33	0.87–2.01	0.18			
**N_stage**
N0	As reference								
N1	1.22	0.89–1.67	0.22						
N2	1.6	1.12–2.29	0.01						
N3	1.61	1.15–2.24	0.005						
**Type of surgery**
Mastectomy	As reference			As reference			As reference		
Non-surgery	1.76	1.43–2.15	<0.001	1.37	1.09–1.73	0.01	1.39	1.1–1.75	0.006
BCS	0.66	0.48–0.91	<0.001	0.98	0.69–1.4	0.93	1.02	0.72–1.43	0.93
**Molecular subtype**
HR–/HER2– (Triple-negative)	As reference			As reference			As reference		
HR–/HER2+ (HER2-enriched)	0.14	0.1–0.25	<0.001	0.34	0.22–0.52	<0.001	0.33	0.22–0.51	<0.001
HR+/HER2– (Luminal A)	0.38	0.28–0.52	<0.001	0.51	0.37–0.7	<0.001	0.49	0.36–0.67	<0.001
HR+/HER2+ (Luminal B)	0.22	0.14–0.33	<0.001	0.19	0.13–0.27	<0.001	0.19	0.13–0.26	<0.001
**Race**
Unwhite	As reference								
White	0.84	0.66–1.08	0.17						
**Marital status**
Married	As reference			As reference					
Single	1.2	0.96–1.48	0.1	1.15	0.89–1.49	0.28			
Unknown	0.89	0.54–1.47	0.65	1.44	0.99–2.09	0.06			

*OS, overall survival; HR, hazard ratio; CI, confidence interval; HER2, human epidermal growth receptor 2; BCS, breast-conserving surgery; IPTW, inverse probability propensity score weighting*.

**Table 5:** Multivariate analysis of prognostic factors of BCSS for specific sites of metastases after IPTW.

**Metastatic sites**	**Only_bone**			**Viscera**			**Bone+viscera**		
	**HR**	**95% CI**	***P-value***	**HR**	**95% CI**	***P-valu*e**	**HR**	**95% CI**	***P-value***
Age	1.01	0.99–1.02	0.14	1.01	0.99–1.02	0.06	1.01	0.99–1.02	0.11
**Grade**
I–II	As reference			As reference			As reference		
III–IV	1.36	1.09–1.69	0.005	1.66	1.21–2.27	0.002	1.92	1.34–2.73	<0.001
**T_stage**
T1	As reference			As reference					
T2	1.54	1.04–2.27	0.03	1.2	0.79–1.8	0.39			
T3	1.91	1.25–2.89	0.003	1.22	0.79–1.88	0.38			
T4	1.76	1.17–2.67	0.007	1.48	0.95–2.33	0.08			
**N_stage**
N0	As reference								
N1	1.22	0.87–1.7	0.25						
N2	1.63	1.12–2.38	0.01						
N3	1.63	1.15–2.3	0.006						
**Type of surgery**
Mastectomy	As reference			As reference			As reference		
Non-surgery	1.69	1.37–2.09	<0.001	1.33	1.04–1.69	0.02	1.38	1.09–1.75	0.007
BCS	0.62	0.44–0.87	0.006	0.89	0.61–1.31	0.56	1.03	0.63–1.69	0.9
**Molecular subtype**
HR–/HER2– (Triple-negative)	As reference			As reference			As reference		
HR–/HER2+ (HER2-enriched)	0.13	0.1–0.25	<0.001	0.34	0.22–0.53	<0.001	0.28	0.16–0.49	<0.001
HR+/HER2– (Luminal A)	0.37	0.27–0.51	<0.001	0.53	0.38–0.74	<0.001	0.37	0.22–0.63	<0.001
HR+/HER2+ (Luminal B)	0.2	0.13–0.31	<0.001	0.19	0.13–0.28	<0.001	0.21	0.12–0.37	<0.001
**Race**
Unwhite	As reference								
White	0.87	0.67–1.12	0.28						
**Marital status**
Married	As reference			As reference					
Single	1.21	0.96–1.62	0.1	1.18	0.9–1.55	0.22			
Unknown	0.92	0.54–1.57	0.76	1.52	1.03–2.23	0.03			

*BCSS, breast cancer-specific survival; HR, hazard ratio; CI, confidence interval; HER2, human epidermal growth receptor 2; BCS, breast-conserving surgery; IPTW, inverse probability propensity score weighting*.

## Discussion

In this large population-based cohort study, the role of surgery for patients with BC remained ambiguous, with no consensus; hence, we employed the SEER population database from 2010 to 2015. We find that the BCS and mastectomy group (surgery groups) had a better prognosis than the non-surgery group. Furthermore, we find that a personalized scheme for *de novo* stage IV BC surgery can be based on different metastatic patterns. Our study shows that BCS offers a significant survival improvement over mastectomy for patients with bone-only metastasis, but not for those with other metastatic patterns. For the first time, this is the largest population-based study to compare survival rates between non-surgery, BCS, and mastectomy individuals with *de novo* stage IV BC.

In our study, surgery was linked to improved BCSS and OS, which were objective, credible, and accurate indexes for patients with BC. *L*og-rank test analysis uncovered significant improvements in BCSS and OS in surgery groups, but not in the non-surgery group. To reduce estimation bias and then study further the efficiency of surgery on BCSS and OS in individuals with stage IV BC, multivariate Cox regression and IPTW analyses were conducted. After adjusting and balancing demographic, clinicopathologic, and therapeutic variables by weighing inverse propensity scores, we found that surgery could prolong BCSS and OS. A previous study based on the SEER database (1998–2011) suggested a survival benefit with a surgical procedure (median OS, 34 months for surgery vs. 18 months for non-surgery), but the data about HER2 status in this study was incomplete (24). However, other recent studies based on the SEER database (2010-2015), the information about HER2 status was integral, also proposed that surgery could improve OS and BCSS in patients with stage IV BC (25, 26). Moreover, one research based on the NCDB database also highlighted that surgery could benefit patients with stage IV BC. In this large cohort, an improved OS was found in the surgery group compared with the non-surgery group even after propensity score matching (HR = 0.68, 95% CI [0.63–0.72], p < 0.001) (27). The above-mentioned findings are in consistent with the previous studies showing that surgical procedure has a key role in *de novo* stage IV BC therapy (17, 25, 26, 28, 29) as surgery may substantially reduce overall tumor burden and improve survival by activating immune responsiveness (30, 31). But other studies held different opinions. A retrospective control study from Massachusetts General Hospital demonstrated no difference in survival between the surgery group and non-surgery group (median OS of 2.4 vs. 2.36 years). The researchers considered that this conclusion was correlated with lead-time bias. Meanwhile, a case-matched study suggested that survival was similar between the above-mentioned groups. So the results were potentially confounded by selection bias and system error.

Due to these biases, randomized clinical trials were designed. MF07-01 trial was a prospective, multicenter, randomized trial to figure out the impact of breast surgery on the prognosis of patients with *de novo* stage IV BC (11). In this study, one group received surgery plus systemic therapy after primary surgery and the other group only received systemic therapy. Surgery might not obtain a survival advantage after 3 years of follow-up, but after 5 years of follow-up, patients receiving surgery could attain a better prognosis. However, TATA, TBCRC 013, and POSYTIVE clinical trials suggested that surgery had a similar prognosis in patients with *de novo* stage IV BC compared with non-surgery (12, 13, 32). Moreover, the Eastern Cooperative Oncology Group (ECOG) 2018 suggested that there were no statistically significant differences in OS and progression-free survival (PFS) between the surgery and palliative groups, while the rate of local recurrence was significantly higher in the palliative care group than in the surgery group (3 year recurrence rate 25.6% vs. 10.2% in the surgery group) (33). In addition, the SUBMIT study (NCT01392586) is a randomized clinical trial that could provide evidence about the impact of surgery in patients with BC with metastatic disease, but it was stopped because of low accrual rate (34).

Based on BC heterogeneity, previous studies have been inconsistent. Past studies have proposed that different metastatic patterns have different biological effects on BC and prognoses may differ with metastatic pattern (17, 35, 36). It was recognized that the most frequent metastasis sites are bones, viscera, and bone+viscera. Bone-only metastases are most common and have the best prognosis (18, 37, 38). These reports were mirrored in our study, where 45.4% of the cohort had bone-only metastasis at primary diagnosis and had a 59.4% survival rate, which was higher than in other groups (viscera: 51%, bone+ viscera: 43.3%). Metastasis site is influenced by BC subtype (39, 40). For example, despite aggressive systemic treatment, HER2-positive, as well as triple-negative, cancers have a high risk of visceral metastasis, while luminal A tumors tend to metastasize to bones (37, 41). We find that 55% of the cohort with luminal A BC had bone-only metastasis, 47% of the HER2-enriched BC cohort, and 48.7% of the cohort with triple-negative BC had viscera metastasis.

Some SEER-based studies suggest that BCS plus radiotherapy had a better prognosis in contrast with mastectomy (42–44), while these studies were conducted on patients with early-stage BC. However, we found that BCS was equally remarkable for individuals with BC, with bone-only metastasis, because patients with BC with bone-only metastasis received radiotherapy or radiotherapy plus chemotherapy were highest among our cohort. Furthermore, relative to mastectomy recipients, BCS may have cosmetic benefits and is safe, and decreases anxiety, psychological morbidity, and depression, improving body image and self-esteem (45–47). In our cohort, the median BC survival time for individuals with BC, with bone-only metastasis, was 31 months, higher than in the viscera and bone+viscera metastases group (both 25 months). Thus, the absence of breasts after a mastectomy had a remarkable influence on the QOL of patients, all the time reminding them that they are patients with BC. Thus, BCS may be recommended for individuals with BC with bone-only metastasis, which provides considerable survival benefits and is more acceptable to patients.

Interestingly, BCS had similar effects on OS and BCSS in patients with viscera and bone+viscera metastases relative to mastectomy, even after combined COX multivariate proportional hazard and IPTW analyses. Breast subtypes were correlated with the choice of surgery type (48, 49). Luminal A and luminal B BC are linked to good prognosis, while Her2-enriched and triple-negative BC have a poor prognosis (37, 40, 50, 51). Meanwhile, the prognosis of patients with bone metastasis was significantly better than that of patients with viscera or bone+viscera metastasis. Herein, patients in the viscera metastasis group had 13.1% Her2-enriched and 10.7% triple-negative, patients in bone+viscera metastasis group had 20.3% Her2-enriched and 24.3% triple negative, and the above-mentioned two groups were both more than bone-only metastasis group. Because patients without bone-only metastasis had shorter survival, BCS had a limited impact on our analysis. Furthermore, individuals with hormone receptor-positive tumors are sensitive to endocrine treatment, while those with HER2-enriched or triple-negative BC lack effective therapeutic targets (52, 53). Meanwhile, we also compared prognosis among three surgery methods based on different molecular subtypes. BCS recipients had similar OS and BCSS relative to mastectomy recipients, but non-surgery patients had a worse effect, regardless of subtype (Supplementary Tables 1, 2). Due to the limited number of patients enrolled in our study, subgroup analysis of metastasis type and molecular typing could not be carried out simultaneously.

Moreover, despite different metastasis patterns, the tumor grade and the molecular subtype were prognostic factors that influenced the survival of patients with *de novo* stage IV BC. Meanwhile, age at diagnose was significantly correlated only with better OS and the threshold value of BCSS. The above-mentioned results were consistent with previous studies investigating prognostic factors in metastatic BC (36, 54, 55). But tumor T stage and tumor N stage had only impacted the patients with bone metastasis in our study. BC was a systemic disease, which had different tumor burdens depended on different biological characteristics. In our study, the absolute survival benefit was observed for women with small primary breast tumors as previous meta-analysis and retrospective study reported (56, 57) because patients with a lower disease burden could have greater benefit from surgery. Patients with a higher disease burden could have had more challenging local control and may have done poorly on this basis. We hypothesized that the size of the primary lesion and the number of lymph node metastasis in patients with stage IV BC with bone metastasis had a great impact on the systemic tumor burden of the patient. Surgical resection could reduce the burden of the local tumor to improve OS and BCSS. But for patients with visceral or multiple metastases, local lesion size and number of lymph node metastasis had little influence on systemic tumor burden, local surgery had a limited impact. Thus, the notion that the T stage and N stage in the stage IV setting could impact survival is plausible, especially in patients with bone metastasis.

This was a comprehensive study of how the benefits of different surgery types vary by metastatic pattern in stage IV BC. However, it has some limitations. First, in our studies, patients needed to be randomized into different groups according to the treatment. Retrospective studies could not be the cause and may be influenced by selection bias and uncontrolled confounding factors, especially metastatic site and non-surgical treatment, even with IPTW administration. Second, due to the lack of information on endocrine, anti-HER2, denosumab or zoledronic acid therapy, family history, patient anxiety, BRCA gene status, and other variables in the SEER database, we were unable to control for these potential modifiers. These factors greatly influence clinical decisions and even prognosis. Third, there was a big gap among the three groups, which may introduce bias to the data, and the sample size was not sufficient to uncover modest differences. Fourth, the SEER data resource only contained data on four site-specific DS sites at primary diagnosis. Thus, we could not obtain details on other DS sites. Lastly, *p* < 0.05 was statistically significant, and the chance of falsely rejecting a null hypothesis may exceed 0.05.

## Conclusion

Our research show that survival benefit from the type of surgery used on *de novo* stage IV BC differs by metastatic pattern. Local surgery for individuals with DS offered a remarkable survival advantage in contrast with non-surgical management, and BCS is the top selection for individuals with bone-only metastasis. Surgical decisions on patients with *de novo* stage IV BC should be customized to metastatic profile. The mechanisms underlying bone, viscera, bone+viscera, or first BC metastasis need investigation.

## Data Availability Statement

Publicly available datasets were analyzed in this study. This data can be found here: https://seer.cancer.gov.

## Author Contributions

KL and CZ drafted the manuscript and analyzed the data. YY, LN, and WZ generated the figure. BW performed the background research. GG and JH edited the manuscript. All authors have read and approved the content of the manuscript.

## Funding

This study was supported by the Health Research Fund of Shaanxi Province (HRFS 2018007 to GG).

## Conflict of Interest

The authors declare that the research was conducted in the absence of any commercial or financial relationships that could be construed as a potential conflict of interest.

## Publisher's Note

All claims expressed in this article are solely those of the authors and do not necessarily represent those of their affiliated organizations, or those of the publisher, the editors and the reviewers. Any product that may be evaluated in this article, or claim that may be made by its manufacturer, is not guaranteed or endorsed by the publisher.
